# Hamitic race theory and African cattle classification, 1868–1971

**DOI:** 10.1080/00033790.2025.2449858

**Published:** 2025-02-02

**Authors:** Tad Brown

**Affiliations:** Department of History and Philosophy of Science, University of Cambridge, Cambridge, UK

**Keywords:** Pastoralism, social theory, cattle, science, race

## Abstract

From the late nineteenth century, European ideas about African cattle breeds relied on the racial classification of African peoples, routed through Hamitic theory. As it were, anthropology influenced the reconstruction of cattle history, and the study of cattle breeds affected perceptions of race. The methods employed to classify African cattle included a range of sources with regards to past human migrations. Through the work of Hellmut Epstein, I detail how the Afrikander cattle breed was seen to signify the spread of an ancient white cultural influence in Africa. Comparisons from West Africa eventually challenged the historical premise of the breed’s classification.

There is no more vital aspect of anthropology than the study of domestic animals.

Raymond Dart (1933)^1^

## 1 The origins of *The Origin*

In 1971, Hellmut Epstein (1903-1987) published a defining work for the field of livestock science, *The Origin of the Domestic Animals of Africa*. His colleagues in South Africa were citing the book as forthcoming in the early 1930s. The time to completion reflected its scope, as the two-volume compendium covered live-stock from all of Africa.^2^ When eventually in print, Epstein thanked Ian Mason at the Institute of Animal Genetics in Edinburgh, who had revised the text and ‘guided the author away from the pitfalls of some long-held but mistaken pet ideas’.^3^ This article focuses on one of these ideas. It revisits Epstein’s conviction that the Afrikander cattle breed was a direct descendant of ancient Zebu herds introduced into East Africa by Hamitic nomads.^4^

**Figure 1:**
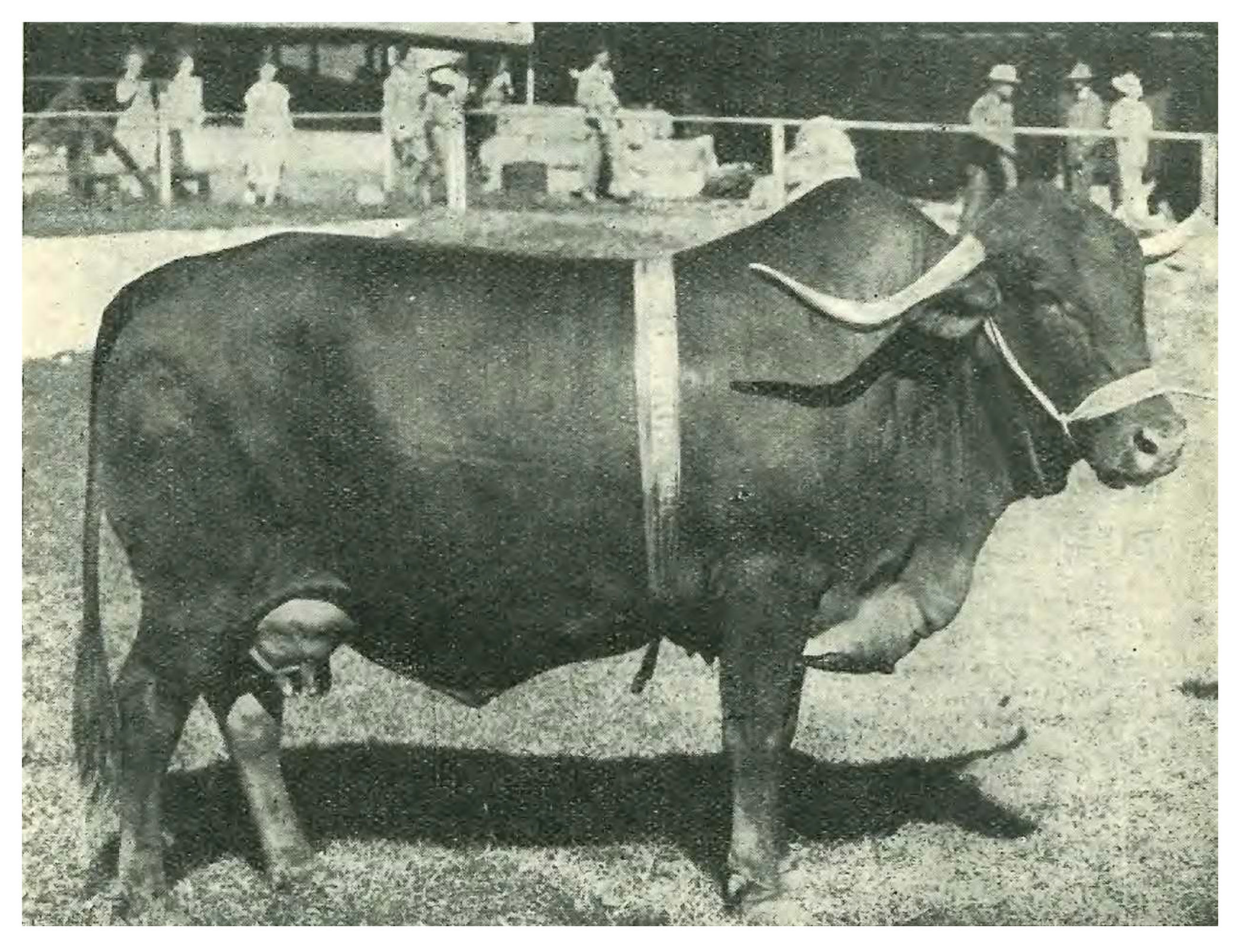
Afrikander cow. Source: N.R. Joshi, E.A. McLaughlin, and Ralph W. Phillips. *Types and Breeds of African Cattle*. FAO Agricultural Studies, No. 37 (Rome: Food and Agriculture Organization of the United Nations, 1957), p. 274. Courtesy of *Farmer’s Weekly*. Reproduced with permission.

Typical descriptions of Afrikander cattle refer to its red colour, sweeping lateral horns, and humpback profile [See Figure 1]. When Epstein ‘took up stock farming in the Western Transvaal’ in 1927, he ‘became interested in the history of the Afrikander breed’ and wanted to know more.^5^ How did these cows come to exist in South Africa? Epstein would identify Afrikander cattle as the lineal offspring of ‘Hottentot cattle’, or cattle owned by ‘Hottentot’ people, a derogatory period term that Europeans applied to Khoekhoe pastoralists in southern Africa.^6^ This anthropological finding inaugurated a lifelong project which culminated in Epstein’s opus with Mason. So, although obscured by the book’s exhaustive content, Afrikander cattle prompted *The Origin*.

This article takes its inspiration from scholarship in animal history on the topic of race.^7^ Jonathan Saha has advocated for studying the ‘relationships *between* species’ to recover the experiences of colonial subjects.^8^ Such analysis provides insight into the encompassment of power, but its focus can inadvertently overlook differentiation *within* species that has routinely been used to define relationships between them.^9^ Colonial scientists drew from human history when representing different breeds of cattle and projected their racial prejudices on to the animals kept by different social groups. Into the twentieth century, Europeans put forth classifications for African cattle that relied on the presumptive origins of African herders: The who, when, and where of African peoples amounted to the what of African cattle.^10^ Epstein’s work offers an interesting opportunity to explore how scientific distinctions between breeds also factored into the construction of race.

In this article, I demonstrate how the study of migrations, anatomy, and languages contributed to the making of divisions within and between humans and cattle. Hamitic race theory will be summarized first, before explaining how Khoekhoe pastoralists and their livestock were fitted into it. I will then review how Epstein classified Afrikander cattle according to the breed’s alleged deep cultural history. The second half of the article compares Epstein’s ethnological inferences about cattle in South Africa with similar questions he then pursued for understanding cattle in West Africa. Theories about the ancestry of Fula pastoralists in West Africa relied on linguistic comparisons and various aspects of physical appearance and cultural practice, including cattle type, to speculate on their origins. In trying to equate a racial affinity between certain herders and their herds, the scientific study of African cattle diversity drew support from the Hamitic hypothesis, only to expose its false suppositions.

In *The Origin*, Epstein conceded that the Afrikander should be classified with other African cattle derived from crossbreeding between the two subspecies of domesticated cattle – *Bos indicus* and *Bos taurus*. As such, the uniqueness he conferred on the Afrikander breed earlier in his work did not hold. This history presents an interesting challenge for scientific writing. Animal geneticists regularly cite Epstein as standard background material in contemporary studies on African cattle diversity without giving context to his claims. Here, I attempt to historicize the development of Epstein’s thinking in relation to the literature from which he drew.

## 2 Noachian casting

Epstein’s interest in Afrikander cattle led to a series of questions, which, as he explained in the preface to *The Origin*, prompted a survey of African peoples. This omnibus approach resembled practices commonly associated with earlier periods of natural history yet continued with regards to cattle in Africa.^11^ At first, Epstein found that there was not a great deal to learn about the Afrikander breed from published materials. A parallel exists with his own early life story. The historical record shows that Epstein completed a doctoral thesis in 1928 from the Agricultural University of Berlin. In the thesis, he debated whether estate trusts could oblige inheritors to improve the conditions of landed property as a legal requirement of title transfer.^12^ Themes of cultural inheritance would become a hallmark of Epstein’s position on *Bos* diversity in Africa.^13^

Long before Epstein, Europeans responded with awe at cattle encountered on the African continent. ‘At the present-day various travellers have noticed the differences in the [cattle] breeds in Southern Africa’, Charles Darwin wrote in 1868. He went on to state that one such traveller

several years ago remarked to me that the cattle possessed by the different tribes of [Africans], living near each other under the same latitude and in the same kind of country, yet differed, and he expressed much surprise at the fact.^14^

For Darwin, social preferences governing which animals were kept and which were killed gave rise to African cattle breeds, without other forethought.^15^ In contrast, he credited conscious selection for the formation of European breeds.

This intellectualist divide made by Europeans was, in part, drawn from a perceived overidentification of Africans with their cattle.^16^ Travellers to southern Africa described Khoekhoe pastoralists as ‘human beasts’ with depictions of living like cattle: That Khoekhoe occasionally draped their bodies in cattle entrails and practiced insufflation (to stimulate milk flow by blowing air into a cow’s vulva) disgusted European propriety and led to totalizing statements about their laggard place in schemes of cultural evolution.^17^ Such portrayals cast Africans with animality, while denying anything of the sort for white observers, a common trope in the history of anti-Black racism.^18^ In fact, Carl Linnaeus had initially assigned ‘Hottentots’ to the species *Homo monstrosus* based largely on the comparative anatomy of genitalia.^19^ European obsession with African sexual organs – both male and female – involved a more general application of social theory, whereby physical stereotypes were used to explain cultural differences.^20^ The established habit of racializing conspicuous body parts also surfaced in Epstein’s theories about African cattle.

Like many of his peers in the early twentieth century, Epstein subscribed to a framework now known as Hamitic theory, which attributed cultural advances in Africa to the conquest of an ancient white race. The biblical etymology of Hamites came from Ham, the exiled son of Noah. Theologians in the Middle Ages had imagined that each of Noah’s sons occupied a landmass and, in doing so, linked Africa to Ham. Proponents of slavery referenced the inescapable curse on Ham’s lineage to justify African enslavement. However, the correspondence between Hamites and Black Africans (then labelled ‘Negroes’) underwent a major reversal in Western ideology. With Napolean’s invasion of Egypt in 1798, European scholars concluded that ancient Egyptians were not as Black as previously believed and attributed their cultural *progress* to Hamites, the white progenitors of African civilization.^21^ In the shifty mishmash of biblical history and emerging race science, Hamites were sometimes, but not always, associated with Semitic peoples.

Historians of science Michael Robinson and Robin Derricourt have both authored definitive accounts of how European explorers and scholars superimposed physical and cultural traits in support of Hamitic race theory.^22^ Its adherents viewed certain features in African people as more ‘European-like’ and applied the term ‘Hamitic’ to describe them.^23^ The ur-narrative was curated from a boggling inventory of sources – linguistic comparisons, cranial measurements, artefacts – and implicated in violent ethnic conflicts across the twentieth century, including the genocide in Rwanda. While rife with inconsistencies, Hamitic theory at its most basic formulation ‘postulated the ancient migration of a “superior race”’ by typecasting African groups through recourse to arbitrary differences.^24^

The nineteenth-century German Egyptologist Richard Lepsius introduced the concept of a Hamitic language family to locate the lost origins of civilization in Africa. He professed that the ancient Egyptians had spoken a Hamitic language.^25^ Philologists believed that the study of unwritten African languages disclosed hidden affinities between cultural groups. Yet what started as the classification of languages soon assumed a racial profile. Physical anthropologists divided human geography based on both physical and linguistic traits, with recognition of a Hamitic type.^26^ The anthropologist Guiseppe Sergi, best known for his use of craniometry in racial classification, was largely responsible for popularizing ‘Hamite’ as a term for certain African peoples – with an Eastern Branch and a Northern Branch unified by a shared skeletal morphology.^27^ This idea that Hamitic-speakers throughout Africa derived from common stock, in both culture and blood, became translated into typological differences between African cattle.

The equation of language, culture, and race was an oversimplified convention with immense explanatory power. Peter Rohrbacher has argued that the rise and fall of Hamitic theory followed that of European colonialism and should serve as a caution to the correlative methods of ethnology.^28^ One linguist went so far as to write that the conflation of subject matter in Hamitic theory ‘at its worst results in treating cattle as a linguistic trait’.^29^ The influence of Hamitic race theory on animal science was not metaphorical. The longhorn *Bos taurus* of ancient Egypt were commonly called ‘Hamitic Longhorns’.^30^ More than shared terminology, African cattle served as a key example within the historical discourse of white superiority.

## 3 Cattle conscripts

In *The Origin*, Epstein identified four basic types of domesticated African cattle and divided these into two main groups. The first pairing belonged to the group *Bos taurus*. That is, both cattle types were humpless, having a body profile with a smooth top-line, separable primarily by body size and horn length. For Epstein, archaeological evidence and depictions in wall art affirmed that long-horn *Bos taurus* – referred to as Hamitic Longhorn – existed in Egypt before 4000 B.C., only to be followed by the smaller Shorthorns (brachyceros) into Lower Egypt over two millennia later.^31^ The delayed arrival fed into the proposal that shorthorn taurines had displaced pre-existing longhorn herds in Egypt, a clue pertinent to Epstein’s argument that Hamitic Longhorns were relatively weak bovines.^32^

Epstein’s second grouping consisted of *Bos indicus* cattle, distinguishable from *Bos taurus* by a hump, certain bone features, large dewlap, and droopy ears. The basic type was known as ‘Zebu’. (Buffon adopted the term after hearing it spoken about a humpbacked cow from India at the Paris Fair of 1752.^33^) According to Epstein, tomb paintings and dynastic inscriptions provided a strong basis for placing Zebu cattle in Egypt circa 1500 B.C, likely as an oddity of pharaonic amusement.^34^ He was not alone in questioning whether these humpbacked animals came to Africa across the Sinai Peninsula – same as *Bos taurus –* or arrived in Egypt by way of Abysinnia (Ethiopia) to the south.

Classification within *Bos indicus* was complicated by crossbreeding.^35^ Epstein used the term ‘Sanga’ for the fourth type of African cattle, those humped breeds that originated from Zebu herds mixing with taurine stock. Historically, Sanga would have been possible once both *Bos taurus* and *Bos indicus* populated the same whereabouts, even if, as conveyed by Darwin’s quote above, cattle in the same general territory could be subject to social circumscription. Authorities debated how Zebu got to Africa, yet most believed that crossbreeding later altered the continent’s entire cattle landscape.

Epstein pondered an alternative version of history. Early in his career, he amassed evidence from the archaeological record to suggest that longhorn Zebu had inhabited East Africa ‘from about 2000 B.C. onwards’.^36^ Whereas textual sources were lacking for the introduction of these longhorn Zebu cattle, historical evidence documented the regular delivery of shorthorn Zebu cattle from across the Arabian Sea to East Africa after 669 A.D., leading to its proliferation throughout the region.^37^ Epstein called the shorthorn Zebu a ‘comparative new-comer in Africa’ and relegated it to a derivative type belonging to Asia.^38^ He was convinced that the Afrikander breed represented a lineage of longhorn Zebu which preceded this more recent history.

According to Epstein, existence of crossbred cattle in South Africa depended on the chronology of migratory events. ‘The dating of the evolution of the Sanga cattle’, he wrote in *The Origin*, was ‘a problem closely connected with the origin and dispersal of the Bantu [people], who were the most important agent in the diffusion’ of the cattle.^39^ The migration of Bantu-speakers to southern Africa circa 700 A.D held a deterministic detail for his equating human races and cattle types. If the influence of *Bos taurus* occurred through the introduction of Sanga cattle and, for Epstein, these Black farmers brought Sanga, the presumption followed that earlier herds in the region must have possessed a different pedigree. Epstein’s classification of Afrikander cattle as a straight Zebu breed was underscored by this transspecies identity claim.

## 4 Oral reconstruction

To understand the breed history of Afrikander cattle, Epstein needed to learn how cattle got to Transvaal in the first place. The record of material remains and the relative dating of cattle paraphernalia, such as rock drawings and ancient art, led archaeologists to conclude that domesticated cattle had been herded to southern Africa. So, inquiry into the origins of Afrikander cattle became a question of who brought herds from elsewhere.

The history of migrations provided a basis from which Europeans deduced other aspects of African history, including differences between cattle. In *The Native Races of South Africa* (1905), the geologist George Stow repositioned Khoe- and Bantu-speakers as emigrants to the region, in that order, laying groundwork for the argument that successive displacements justified white nationalism.^40^ Further distinctions between people mattered in his work. Stow considered Korana nomads as that Khoekhoe branch ‘able to preserve a more consecutive history’ than any other Khoekhoe.^41^ Superlatives aside, the individual memories expressed by Korana during the colonial period shed light on indigenous ideology and social life in the Cape.^42^ Hamitic theory presented a unifying framework for reconstructing history through the comparison of such cultural details. In this case, the ethnographic record already contained a direct statement on Khoekhoe origins.

An early Dutch missionary to the Vaal, a Mr. Kallenberg, recorded Korana oral history which stated that their ancestors fled from the lake country of eastern Central Africa (late 1500s A.D.) and trekked west – with their cattle herds – to the Atlantic coast, then down to the Cape.^43^ Epstein recruited this testimony as proof of his thesis and, while questioning the accuracy of its timeline, found it obvious that Khoekhoe preceded all but ‘Bushmen’ to the Cape, the latter not owning cows and speaking a separate language.^44^ In this regard, Epstein was enrolling Korana teachings into his own models of race and breed history.

Historians who have studied the use of indigenous knowledge as credible evidence in science highlight its selective translation and complicated coloniality.^45^ Such a conclusion can be drawn from Epstein’s analysis as well, which raises important questions as to how his interpretations have been taken up into broader discussions of African history. For example, John Lamphear recounted the significance of a grey bull in Turkana origin stories to argue that historical scholarship underestimates the realism of oral traditions.^46^ For Lamphear, the grey bull indicated a particular kind of animal rather than a mere synecdoche of Turkana corporate identity: The grey bull symbolized the acquisition of shorthorn Zebu cattle in East Africa. Interestingly, Lamphear cited Epstein to support this interpretation, suggesting that Turkana resistance to the adoption of shorthorn Zebu came from a cultural predilection for the original longhorn Zebu of Africa.^47^ In this way, the idea that Afrikander cattle dated to an unbroken lineage from East Africa has directly influenced African historiography.

Epstein’s views on Khoekhoe cultural memory suited his idea ‘that the Hottentots obtained their cattle and sheep directly from Hamites or Semites’, who had entered East Africa with herds of longhorn Zebu.^48^ The proposed route of ingression proved crucial to the theory portraying Afrikander cattle as an intact Asiatic lineage.^49^ His logic was once again easy to comprehend. If a vanguard of nomads with longhorn Zebu sojourned southward before Bantu-speakers, and no cattle were in the region beforehand, these herds would be the original cattle of South Africa. Nonetheless, the sheer passage of time posed a problem that Epstein needed to address. European sailors rounding the Cape of Good Hope had witnessed cattle ashore in the late fifteenth century, and notebooks from the arrival of the Dutch East India Company to the Cape in the 1650s describe huge Khoekhoe herds resembling Afrikander cattle.^50^ Armed with this knowledge, Epstein insisted that Khoekhoe had experienced negligible contact en route to the Cape, thereby protecting their longhorn Zebu herds from any ‘foreign influence’.^51^ This version of history conveys a noticeable favouritism. Epstein was of the opinion that ‘most Bantu [cattle] breeds are small and of poor appearance’, or clearly inferior to Afrikander.^52^

Epstein likewise refuted the prospect of crossbreeding in the make-up of modern Afrikander herds. Early cargo shipments to the Cape included live cattle, yet certain Boer families had refused to let imported bulls mate with their cows, a foreshadowing of the racial anxieties under apartheid.^53^ In fact, Afrikander breed purity obtained mythic status in Afrikaner nationalist narratives of South African history.^54^ When Boer pioneers left for the interior after 1836, known as the Great Trek, the oxen yoked to their wagons became celebrated as Afrikander. This tendency became pronounced following the Anglo-Boer War (1899–1902), during which the breed was known to have suffered many losses.^55^ Subsequently, the Afrikander Cattle Breeders’ Society formed in 1912 to promote studbook registration and set standards of excellence for the breed.^56^ This is the context in which Epstein argued that on the veld of South Africa grazed an uninterrupted line of purebred longhorn Zebu, similar to those ‘brought by Semitic nomads from Abyssinia’ some 3,500 years ago.^57^

## 5 Race science meets cattle classification

A year prior to departing from Transvaal in 1934, Epstein published an article in the *Journal of Heredity* which introduced his theory of Afrikander cattle origins. He identified East Africa as the genesis of Khoekhoe pastoralists and the whereabouts of their encounter wth incoming Hamites, an understanding owed to Charles Seligman, a key figure in British ethnology.^58^ Seligman established the Hamitic influence through the study of history, migration, social customs, and human biology.^59^ His works were immensely influential for the overarching narrative of Hamitic race theory. For instance, Seligman concluded that veneration towards cattle in Africa was a cultural trait obtained through an ancient interaction with Hamites.^60^

Authors in the early nineteenth century had ascribed ‘Hottentots’ and ‘Bushmen’ to the same racial provenance. Neither group was viewed favourably by Europeans, but a distinction emerged, at least in English literature, in which a greater moral character became attributed to the property-owning pastoralists.^61^ Cultural habits separated the two, with livestock as the decisive factor. Through Seligman, Epstein inferred that Khoekhoe were ‘one of the most southern African races of partly Caucasian origins’, whose ancestors had, as evidenced by their Zebu cattle and fat-tailed sheep, experienced cultural contact with Hamites.^62^ The most bracing Hamitic detail, however, was a racial contrast with other Africans.

The identification of separate peoples in South Africa was made to accommodate popular notions of cultural evolution. As Raymond Dart, professor of anatomy in Johannesburg, wrote,

these three groups of African peoples – Bushmen, Hottentot, and Bantu – have been sufficiently conservative to present us with a living picture of the three fundamental phases or strata in the passage of mankind from the state of Nimrod [grandson of Noah] to that of ancient Egypt.^63^

For Dart, the serial chronology was accompanied by modes of production: hunting-gathering came first, followed by pastoralism, and eventually agriculture. Scholars relied on biological and cultural comparisons in Africa to estimate ‘the antiquity of prehistoric Man’ and to explain the continent’s historical trajectory.^64^ But really, the portrayal of static culture-types helped to justify violent racial displacement as inevitable.

In *The Origin*, Epstein restated the theory that Khoekhoe in South Africa ‘derived from an early light-skinned Hamitic stock’ or were a ‘Bushman people influenced by Hamitic culture’, which in some remote past had shaped the ancestry of the South African pastoralists.^65^ According to this synopsis, the Caucasian encounter not only supplied Khoekhoe with animal husbandry and cattle veneration, it furnished them with ‘a longer, higher and narrower head, slightly narrower nose, rather more prognathous face, somewhat lighter skin colour, and very different blood grouping’ than other Africans.^66^ Again, Hamitic theory endured by its capacity to construct race based on the overlap between ethnological particulars. The imagery and language of cattle breeds provided extra fodder for upholding racial distinctions.^67^

Just as scientists subsumed racist cultural commentary into the classificatory impulse for humans, physical stereotypes were used to explain differences with cattle. In one influential interpretation, which came through a Germanic scholarly tradition, cattle type served as a proxy of people’s collective identity. Leopold Adametz was a professor of animal studies at the University of Natural Resources and Life Sciences in Vienna. At the 1889 Paris World Exhibition, Adametz saw a cow that resembled the longhorn cattle of ancient Egyptian art.^68^ (The cow belonged to the N’Dama breed, to which I will soon return.) From this, Adametz assumed that pastoral groups had historically migrated with their own livestock breeds; that the same people, even in vastly different geographic regions, possessed a consistent type of cattle.^69^ Epstein would become an Adametz devotee.

Epstein rallied behind osteological findings to identify Afrikander cattle as longhorn Zebu in its purest form. In 1934, he published a paper with H. H. Curson, a South African research officer at the Onderstepoort Veterinary Institute, just outside Pretoria, comparing the skulls of Hamitic Longhorn, West African Shorthorn, and Afrikander cattle. Of all the features to distinguish between cattle types, Curson and Epstein considered the skull as ‘the feature of greatest importance in the study of ancestry’ because its details could be measured with precision. According to the authors, while the exact pedigree of each specimen was unknown, the three skulls exhibited characteristic differences in profile, eye orbital, and horn cross-section.^70^ I will save my larger discussion on the metrological history of cattle craniums for another article. What is important here is that their skeletal study contributed to the ‘racial history of African cattle’ already underway by Epstein.^71^ Reference to race with regards to *Bos* biology, in this case, indicated a former geographical isolation between the main cattle types during their evolutionary history.

A year later, the supposed uniqueness of longhorn Zebu was confronted with an anatomical challenge. Curson had joined with J.H.R. Bisschop, a fellow veterinary officer at Onderstepoort, to review the *Bos indicus* hump. The typical shorthorn Zebu cattle had a thoracic- or chest-hump, given its position on the animals’ back. But the Afrikander cattle exhibited a cervico-thoracic hump, more towards its neck. Epstein regarded the Afrikander neck-hump as a biological adaptation to seasonal scarcity on the steppe of Central Asia.^72^ To him, the neck-hump was the original type and the shorthorn Zebu chest-hump was merely a later modification wrought by the powers of selection in the longhorn Zebu. Curson and Bisschop refuted Epstein on the matter.

When viewed from the anatomy itself, Epstein’s theory that the Afrikander neck-hump derived from life on the Asiatic steppe did not add up. Both the Afrikander and Sanga cattle had muscular humps. Only the shorthorn Zebu hump stored fat. The evidence gave Curson and Bisschop good reason to wonder if the Afrikander breed had resulted from an encounter between *Bos indicus* and *Bos taurus*, similar to that of other Sanga cattle. Epstein denied this conclusion. To him, the internal similarity between Sanga and Afrikander could just as readily have resulted from the parental influence of Afrikander stock in the make-up of Sanga or shorthorn Zebu. Additional methods were needed to settle the debate. In embryology, the ‘doctrine of recapitulation’ stated that unborn foetuses would reveal an identical structure if the animals shared an ancestral history.^73^ A quick response from contacts at the Veterinary Services in Tanganyika affirmed that a premature newborn Zebu displayed the musculature and thoracic position as it would as an adult.^74^ It was nothing like an Afrikander and did not exhibit a neck-hump during its developmental stage.

From all of this, Curson began to wonder about the indefiniteness of the word ‘Zebu’. Dictionary definitions indicated that Zebu were domesticated cattle from the Indian subcontinent, hence the name *Bos indicus*. What about African Zebu? Curson mused, ‘it would seem that one school refers to only Eastern cattle as Zebu, while the second includes all humped cattle whether Asiatic or African’.^75^ His provisional solution to the dilemma was to retain all Zebu of Asiatic origin in one category and demote Zebu originating in Africa to a pseudo-status.^76^ Tellingly, Curson kept Afrikander cattle with the Asiatic class. He seems to have agreed with Epstein in this regard.

To learn more, Curson and Thornton sent a questionnaire to colonial veterinary officials throughout Africa and beyond.^77^ They credited Epstein for the research agenda. ‘It was Epstein’, they acknowledged, ‘who in 1933 first linked the scattered facts and put forward a working hypothesis in regard to the origin of our [Afrikander] cattle, associated with, of course, the various human migrations’.^78^ The authors recited the history that shorthorn *Bos taurus* had entered Lower Egypt with an incoming Mediterranean race and ‘compelled the original tribes (of Negroid stock) to retreat’ westward with their Hamitic Long-horns. The changeover between cattle types occurred over centuries, yet Curson and Thornton noted that it was contemporaneous with the arrival of Semitico-Hamites, who ‘were accompanied by lateral horned cattle of zebu type’.^79^ The Afrikander breed, therefore, represented to them a rare holdover from ancient history, because if not for the pastoral exodus out of East Africa, the longhorn Zebu population of Africa would have all become Sanga as herds interbred.

## 6 An outlier out west

**Figure 2:**
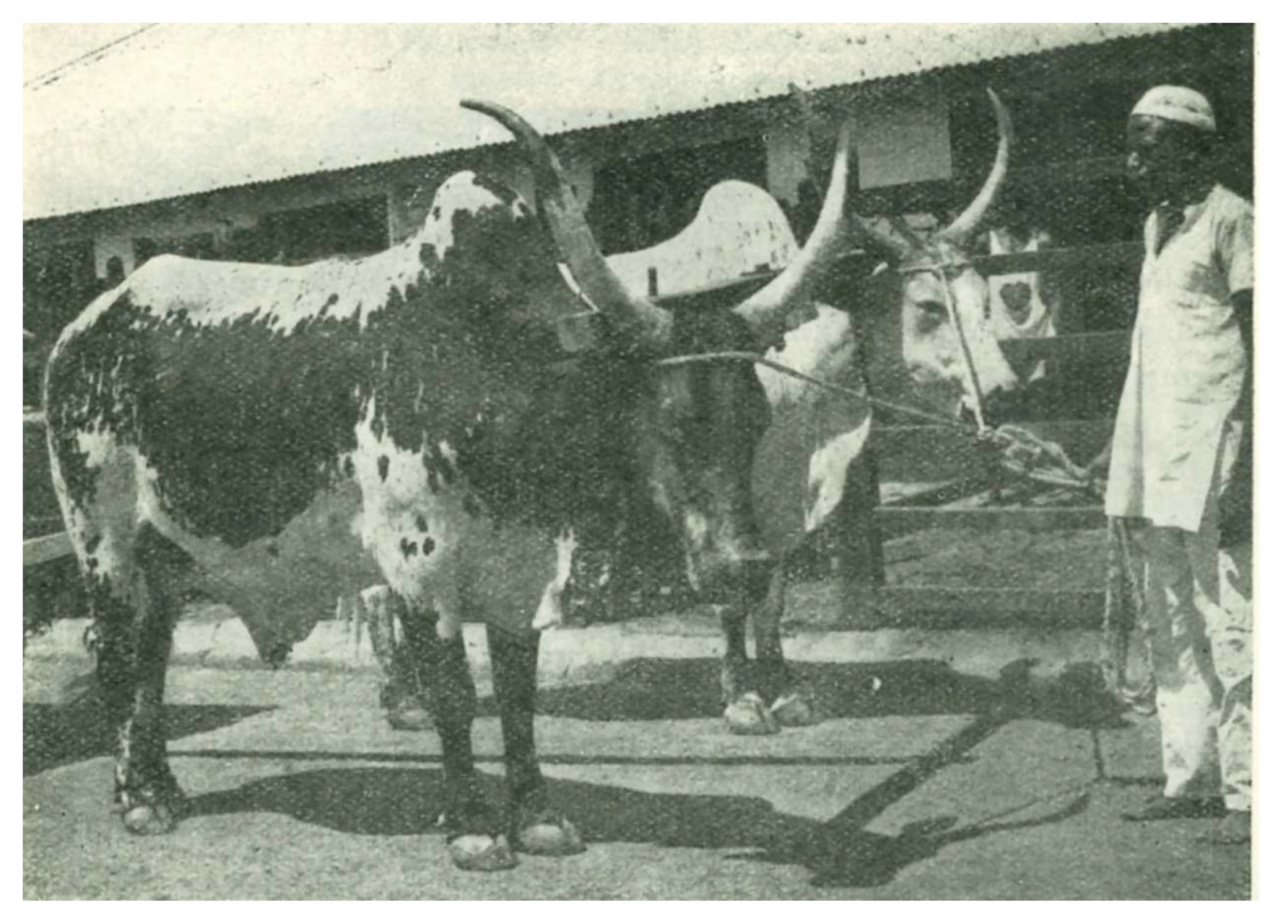
A yoked pair of Lyre-horned White Fulani Cattle with handler, whose name was not reported. Source: N.R. Joshi, E.A. McLaughlin, and Ralph W. Phillips, *Types and Breeds of African Cattle*. FAO Agricultural Studies, No. 37 (Rome: Food and Agriculture Organization of the United Nations, 1957), p. 105. Reproduced with permission.

The survey responses sent to Curson and Thornton forced a reckoning with the current state of knowledge about African cattle. In their classification, all ‘True Zebu’ were Asiatic in origin, which, despite the name, included the Afrikander. Reports from elsewhere on the continent, however, registered a possible exception. There were humpbacked cows with long horns in West Africa, classed by Curson and Thornton as ‘Lyre-horned Zebu’. Representative breeds within the type – White Fulani and Mbororo – posed a blatant challenge to the presumptive cattle history coming from South Africa [See Figure 2]. If Khoekhoe fore-fathers had sourced Zebu stock from Hamitic pastoralists in East Africa, did these analogic cows in West Africa share the same origin story?

In their work, Curson and Thornton chose to refer to the Afrikander as the one and only ‘Lateral-horned Zebu’, within the True Zebu class, and in turn grouped the Lyre-horned Zebu with Sanga cattle under the category ‘Pseudo-Zebu’.^80^ Epstein deemed the choice of terminology as ‘not a fortunate one’ but agreed with their reasoning for it.^81^

Epstein would make a concession of his own with regards to the distinguishing features for Zebu classification. He began to promote the position of the hump, not the size or direction of horns, as the proper convention for grouping within *Bos indicus*. Epstein’s updated rubric divided Zebu cattle between neck- and chest-humped. For him, most true Zebu of Asiatic stock were chest-humped. As to be expected, Epstein identified Afrikander cattle as the ‘one comparatively pure zebu breed of the neck-humped type [which] has survived’.^82^ All the other neck-humped cattle in Africa were taken to indicate crossbreeding between *Bos indicus* and *Bos taurus*. Epstein then motioned to recategorize the Lyre-horned Zebu of West Africa as a ‘Chest-humped Sanga’. It did not, in his estimation, deserve the associated Zebu status that Curson and Thornton had bestowed on the type with the Pseudo-Zebu name.

There was one person who rejected any reference to Sanga cattle in West Africa. That person was Mason, who would later revise *The Origin*. In his 1951 publication *Classification of West African Livestock*, Mason stated that West African cattle were derived from herds ‘coming from, or at least *through* Egypt and via French North Africa’.^83^ Whatever cattle had migrated across the Maghreb long ago would have contained taurine bloodlines. Based on his study, Mason found the use of Sanga to be an inappropriate catch-all category for grouping breed diversity in the region. He also thought that Curson and Thornton had betrayed a basic misunderstanding of the parentage for certain West African cattle.^84^

## 7 Continental cultural connections

No other cattle possessed the Afrikander profile because none shared its unique Hamitic history, or so suggested voices from South Africa. That certain hump-backed cattle in West Africa also had long horns posed a potential problem with this hypothesis. Scientific knowledge of cattle in West Africa was ‘as yet very meagre’ when Epstein set to his task in the 1930s.^85^ Nonetheless, the question of when pastoralists brought cows to West Africa proved consequential to his classification of Afrikander cattle as Zebu proper. As with South Africa, the racialized history of migration again served as an explanatory factor in the identification of African cattle types.

Livestock scientists in Onderstepoort pointed to geography as a good reason for making comparisons between the regions. Curson and Thornton phrased it thus: ‘West Africa represents, as does South Africa, a *cul-de-sac* and it is the furthest point which could be traversed by tribes migrating from the north and east’.^86^ The routes in this cartographic visage – as rays extending out from Egypt – enacted the imagined conquest of Africa in Hamitic theory. In summary, the theory held that lookalike cattle in remote regions shared a consanguineous history if herded by people with similar cultural and racial antecedents.

Veterinary knowledge of disease ecology helped connect cattle across distant geographies. Most importantly, an absence of longhorn *Bos taurus* in twentieth-century Egypt caused scientists to question the possible whereabouts of such cattle or their descendants.^87^ Colonial officers identified ‘a more or less pure [cattle] type along the Gulf of Guinea’ that was similar to depictions of Egyptian stock.^88^ This type, the N’Dama breed of West Africa, fit the general description of animals referred to as Hamitic Longhorn, albeit with a smaller frame.^89^ Recall that a chance encounter with N’Dama had inspired Adametz to formulate his hypothesis that Hamitic peoples retained a cattle type preference. While diminutive in size compared to its predecessors, N’Dama seemed to confirm the idea that pastoralists had departed for the Mediterranean littoral when short-horn taurines entered the Nile Delta.

Archaeological evidence suggested that longhorn *Bos taurus* cattle had dispersed across North Africa and would have continued down around the coast if not for a biting fly. The distribution of tsetse flies, vector of a debilitating cattle disease known as trypanosomiasis, obstructed the equatorial advance of nomadic pastoralists and their herds. Rather than proceed all the way to the Cape, the Hamitic Longhorns got turned around in the cul-de-sac of West Africa.^90^ Pastoralists in East Africa may have been able to drive their herds the distance. Other nomads were not. Colonial reports on N’Dama bolstered Adametz’s idea that collective identity was entrained in animals’ bodies and that migrants had fled ancient Egypt with Hamitic Longhorns first and Short-horns later.^91^

In *The Origin*, Epstein cited the horn length of N’Dama as an indication of its Egyptian origin.^92^ Environmental stressors made for a smaller animal, but otherwise the breed matched ancient descriptions of the earliest domesticated cattle in Africa.^93^ This broader history of Hamitic Longhorns contained useful parallels for interpreting findings in South Africa. According to Epstein, Khoekhoe cattle herds displayed long horns because this was a Hamitic preference obtained from Hamites in East Africa.^94^ Not only did long horns serve as a tether for connecting distant peoples with a shared cultural background, but the racial classification of Africans by Europeans consistently incorporated ideas about the biology of African cattle.

There was a pastoral group in West Africa, known as Fula (*Fulbe*, Fulani) whose zeboid cattle sported long horns.^95^ Seligman assigned Fulani to the Northern Branch of the Hamitic race.^96^ Following suit, Epstein relied on Seligman and the burgeoning ethnological record to complete his survey of African livestock in *The Origin*.^97^ The same sort of inquiry he employed to identify the source of Afrikander cattle returned in trying to comprehend Fula cattle in West Africa. Where did these nomadic herders come from? When did they acquire Zebu? And, perhaps foremost for Epstein, what connections did this imply for the origin and descent of Afrikander cattle? As before, the synthesis of evidence from linguistics, anthropology, and anatomy came routed through Hamitic theory.

## 8 Fula phenotype and pastoral phylogeny

The idea that ancient racial migrations explained existing cattle diversity met with conflicting evidence in West Africa. As mentioned, reference to Hamitic Longhorns came from the Hamitic language family ascribed to an ancient Egyptian race. Various colonial officers in West Africa classified the language spoken by Fula (*Fulfulde* or Fula) as a Hamitic language.^98^ The linguistic grouping helped to explain Fulani racial history and restore lost cultural connections, with a bearing on African cattle classifications. The ability for one body of evidence in Hamitic theory to model another was seemingly endless. Epstein concluded his discussion of humped cattle in *The Origin* with a historical review of Fulani origins and the descent of their long-horned cattle.

By the end of the nineteenth century, the scholarly study of African languages extended from the Bantu languages of South Africa to the Berber branch of the Hamitic family in West Africa. However, the Fula language had yet to attract the same scholarly devotion.^99^ There was no published standard dialect from which to judge vernacular differences as an archive of historical change.^100^ This absence invited other cultural aspects into the debate about Fulani history. One notable French authority on West African peoples proposed an ancestral Fulani exodus from the Near East. M.M. Delafosse gained attention for the hypothesis ‘that certain peoples, whom [Delafosse] calls Judæo-Syrian, migrated from the region of Syria to Cyrenaica at a very remote period. They spoke an Egytpo-Aramaean tongue, and their religion was an early form of Judaism’.^101^ As told, these nomads continued westward across North Africa and into the Upper Niger around 200 A.D., eventually becoming Fula, who self-identified as *Fulbe*. While the specific route of migration was the subject of intense historical debate, the pastoralists’ past had few doubters.^102^

The Fula language posed trouble for linguists because it seemed to share little in common with other languages in West Africa. In the hypothesis by Delafosse, Fulani had lost their language in the process of becoming an ethnic group. Whatever mother tongue the ancient nomads spoke had disappeared without a trace. The possibility of an idiomatic assimilation, where migrants’ way of talking became absorbed into the local language, opened parallel interpretations for other peoples. One scholar at the time surmised that entire languages may have existed in West Africa that were allied ‘to the Hottentot languages of South Africa’.^103^ A proposed grammatical link between Fula in West Africa and Khoekhoe in South Africa reconnected peoples with supposed Hamitic antecedents.

Whatever Aramaean tongue may have been spoken by progenitor Fula left no historical clues. The point was not missed on Henry (‘Harry’) Johnston. A self-taught British ethnographer, Johnston wrote extensive observations on African languages in West Africa.^104^ Epstein would draw from Johnston’s work to juxtapose West Africa cattle with the humpbacked cattle in South Africa. ‘There is no more interesting problem in African ethnology’, Johnston opined in 1921, ‘than the origin and relationships of Fula people and of their language’.^105^ He strongly questioned the Hamitic classification of the Fula language that was gaining credibility in texts. ‘To class Fula in any way with the Hamitic family is monstrously misleading, though it is an error to be found in many superficial writings on African philology’, Johnston noted.^106^ Others questioned his orthography and basis for authority.^107^ Nonetheless, there was a European fraternity with Fula that played into the misleading classification.

Whilst Johnston saw no lexical evidence for including *Fulfulde* in the Hamitic language family, he was decidedly less demure about their ancient racial history. He stated that Fula nomads were ‘unconsciously carrying on the Caucasian invasion’ of Africa.^108^ Such representation was consistent with the Hamitic grouping. The pastoral Fulani fit an epic of conquest that dated back to biblical times, one which European authors longed to uphold. As linguist Joseph Greenberg observed in 1949, the ‘impulse to classify Fulani as Hamitic’ came from an unspoken desire to correlate ‘the conquering-cattle-owning-Hamite’ with a living African archetype.^109^ Language studies failed to retrieve a forgotten Fula passage through ancient Egypt.^110^ Still, Europeans convinced themselves that a deeper genealogy could be read through Fula bodies and herds.

Debates about Fula, with implications for their lyre-horned cattle, became heated in the twentieth century. When Johnston published *The Opening Up of Africa* in 1911, he attributed Fulani origins to a Hamitic exodus from Egypt, not based on linguistics but race.^111^ ‘One of the most remarkable of human elements in the present composition of West Africa and the Sudan, and equally one of the most potent of “white” influences in moulding Negro Africa is and has been the Fula people’, Johnston stated.^112^ To quote him at length:

In appearance the Fula man or woman of pure race is a handsome type of human being. They are tall people, beautifully formed as regards the proportions and carriage of the body, comparable sometimes to the much quoted Greek statue. The appearance of the face frequently recalls the dynastic Egyptian or Pharaonic type, the nose is usually straight and well-formed when there is no strain of Negro blood derived from recent intermixture.^113^

The Hamitic narrative indebted Fulani heritage to a Caucasoid past, for which Europeans found evidence in the pastoralists’ physical features.

Because not all Fula were nomadic in the early twentieth century, observations about their lifestyle gave rise to biological explanations for Fulani cultural heritage. Colonial officers and travellers contrasted the lighter skin complexion of ‘pure’ Fula pastoralist with ‘bastard Fulani’ in settled villages.^114^ Fula history became read through a half-caste racial heritage, one that associated miscegenation with agriculture and general misfortune. Epstein applied this understanding to his livestock studies. He noted that ‘“Cattle Fulani”, who are the purest representatives of the Hamitic element, lay more stress on horn size than do the settled Fulani who are much mixed with negro blood’.^115^ In this way, Epstein assumed that animal conformity to an ideal breed type revealed a racial genealogy, a lasting theme in the Hamitic interpretation of the archaeological record as well as reports on cattle diversity.^116^

Some colonial officers invoked a lost language or physical features to speculate on Fulani history. Others fashioned the past through pastoralism itself. One custom reappeared in accounts of Fula identity that marked a historico-cultural contiguity with ancient Egypt: cattle worship. Commentators interpreted Fulani reverence for cattle as a surviving relic from pagan idolatry, despite their widespread conversion to Islam. British explorer Edmund Morel concluded that Fula ‘customs bear record of their progenitors having been influenced both by the cult of ancient Egypt and by the Israelites’.^117^ The religious sentiment Fula held towards their animals became, in the framework of Hamitic theory, evidence that the same people who had inhabited the Lower Nile went forth to multiply throughout Africa. Scholars related the wanderlust of cattle-rearing to a history of political conquest, whereby Fula livestock wealth symbolized the warlike nature of its herdsman, a Hamitic characteristic borne of seeking pasturage through strength.^118^

At the turn of the century, colonial officers had begun to associate Fula pastoralists throughout West Africa with Zebu cattle. In 1906, a French veterinarian in West Africa published a seminal manuscript that divided the region’s cattle between humped Zebus and humpless taurines.^119^ The pairing of Fula with Zebu furthered the Asiatic theory of Fula origins, as Abyssinian cattle were said by Morel to ‘belong to the same breed’.^120^ Any correlation between Fulani pastoralists and Zebu cattle, however, complicated the chronology of cattle in West Africa. According to Epstein, Fula pastoralists had, ‘in the course of their eastward expansion’ across the region, acquired Zebu cattle sometime after the late 7th century A.D.^121^ Fula cattle consciousness may have dated to a distant migration, but Epstein thought that their humpbacked breeds were of much more recent origin.

The *Bos taurus* that had populated West Africa since the earliest pastoral migrations had long horns but no hump. Such was the bloodline leading to N’Dama. Epstein maintained that Fula had obtained Zebu cattle later in history, and he rejected the purity of type formation in their herds. ‘The origin of the long-horned Fulani cattle of West Africa must therefore’, he pronounced, ‘be regarded as similar to that of the sanga cattle of East and South Africa’.^122^ The Director of Veterinary Services for Gold Coast agreed, stating that the Fulani lyre-horned breeds originated from ‘the fusion of the Zebu proper and the Hamitic Longhorn’.^123^ This made sense if no westward migration of longhorn Zebu had taken place in the distant past. It also held the undoing of Hamitic race theory for Afrikander cattle. The same historical argument could, and would, apply to cattle in South Africa.

Epstein’s feeling that Afrikander cattle were purebred stock from the earliest introduction of long-horned *Bos indicus* to Africa thousands of years ago could not withstand sustained scrutiny. Comparative morphological evidence dislodged his faith in the exceptionalism of a true neck-humped African Zebu. Through Mason it became clear to Epstein that the origin of Pseudo-Zebus in West Africa was likely the same for Afrikander in South Africa.^124^ *Bos taurus* had crossed with chest-humped Zebu at some point to create the distinctive type. Epstein reneged on his ‘pet idea’ in *The Origin*. With a hint of reluctance, he concluded that the Afrikander breed, while ‘derived from the cattle of the Hottentots, are also classed with the sanga group’.^125^ He had been mistaken. The cattle were mixed-up too.

## 9 A curious finale

Historians have stated that certain aspects of ethnic identity in Africa emerged as a negotiated response to colonial hierarchies. An example given by Frank Salamone was that British expatriates in Northern Nigeria singled out Fula as the ruling class. ‘The fact that Fulani claimed non-Negroid ancestry and were Muslims allowed them to be categorized as “true rulers” and natural allies of the British’, Salamone explained.^126^ His argument is concerned not with whether Fulani existed prior to this period but in how ethnic identity became a useful category for the British colonial administration. Manufacturing of opposition between colonial subjects included the racialization among their livestock.

Also lost in analyses that totalize culture groups are the internal dynamics within said groups.^127^ A related point applies for categorizations of cattle. Breed discourse imparts a purity to past populations that did not exist. As shown here, the study and classification of African cattle diversity experienced a ‘South Africanization’ through Epstein and his peers at Onderstepoort, who had initially bonded over the Afrikander breed.^128^ After leaving Transvaal in 1934, Epstein served as a Technical Adviser to the Meat Division for the Government of Palestine, followed by an identical position for the Government of Israel. He went on to retire as Chair of Animal Breeding at the Hebrew University of Jerusalem. His earlier connection with Tranvsaal lasted in at least one manner. Epstein acknowledged the South African Friends of the Hebrew University of Jerusalem for financial support in publishing *The Origin*.^129^

The importance of Afrikander cattle to knowledge about *Bos* diversity in Africa effected a curious reversal in the European order of things. Khoekhoe, as an objectified grouping, ascended the ranks of race science partly through an association of their cattle with nomadic Hamites.^130^ Cattle biology provided cues for reading ideas about whiteness into African history. A category within one species – Hamitic – became a shared racial label by which to project hierarchical relationships between groups from different species. This convoluted process entailed its own reversal. Under the influence of Hamitic theory, so-called Hamitic Longhorns became associated with Africans not considered to be of Hamitic descent.

The coupling of prejudicial ideas about human cultural evolution and cattle origins demonstrates the extent to which Hamitic theory accommodated any and all phenomena. Reference to the Hamitic language group experienced a sharp downturn across the twentieth century, yet the terminology and its inherent conceptual biases endured outside linguistics.^131^ While no reasonable scholar endorses Hamitic theory today, dispelling vestiges of the myth will require that animal scientists be aware of racialized genealogies in the history of African cattle classification.^132^ This is not a call to retroactively cancel Epstein. How his works get cited, however, deserves critical review. Scientific papers that reference Epstein could do better at considering how the concept of human races factored into his livestock studies.

In other ways, Mason seems to have revised Epstein’s opus to good effect. Contemporary studies support the notion that Sanga derived from crossbreeding in East Africa and – based on genetics, geography, morphology, and history – classify Afrikander as a separate breed within the subgrouping of Sanga from southern African.^133^ Archaeologists for their part have continued to appreciate the high mobility of early pastoralists across eastern and southern Africa, while limiting the interpretive overreach from material remains.^134^ There is always something to glean from the ethnological record, but caution applies when resorting to cultural habits to distinguish between both past peoples and past cattle. More work is needed to understand the traffic in methods and models between race science and animal science.

This article has shown the troubled relationship between histories of human migration and the classification of African cattle. It has explained how comparative ideas about ‘Hamitic peoples’ shaped understandings of cattle origins and descent, and, in turn, how historical ideas about African cattle informed anthropological theory. Breed groupings were used to secure pseudoscientific categories of race. A new understanding is gained by resituating the study of cattle classification within this ideational context. Under the influence of Hamitic theory, scholars from various disciplines joined in racializing both humans and cattle on the African continent.

## Footnotes

1 Raymond A. Dart, ‘Studies in Native Animal Husbandry, The Domesticated Animals of Pre-European South Africa’, *Journal of the South African Veterinary Association*, 4.2 (1933), 61–70 (p. 61).

2 Half of the first voume is about dogs. Publishers rejected the manuscript for years due to its copious illustrations, until a subsidy from the South African Council for Scientific and Industrial Research in 1950 offset production costs. A condition of the subsidy was consenting to its revision.

3 H. Epstein, *The Origin of the Domestic Animals of Africa, Vol. I & II*, Revised in collaboration with I.L. Mason (New York: Africana Publishing Co., 1971), p. vi. Some citations list Mason as an author, but Epstein was the sole author.

4 The breed name is also written as Africander, Afrikaner, and Africaner.

5 Epstein, *The Origin*, preface, p. v.

6 The name ‘Hottentot’ was first applied to Khoekhoe people after Dutch inhabitation at the Cape. The category was then extended to include inland peoples with similar language and lifeways. See Nienaber, ‘The Origin of the Name “Hottentot”’, *African Studies*, 22.2 (1963), 65–90. When ‘Hottentot’ is written in this article, it is done so as a reference to the actors’ historical category.

7 For examples, see Mara Dicenta, ‘White Animals: Racializing Sheep and Beavers in the Argentinian Tierra del Fuego’, *Latin American and Caribbean Ethnic Studies*, 18.2 (2023), 308–29; Saheed Aderinto, *Animality and Colonial Subjecthood in Africa: The Human and Nonhuman Creature of Nigeria* (Ohio University Press, 2022); Jules Skotnes-Brown, ‘Domestication, Degeneration, and the Establishment of the Addo Elephant National Park in South Africa, 1920s–1930s’, *The Historical Journal*, 64.2 (2021), 357–83; Gabriel N. Rosenberg, ‘A Race Suicide among the Hogs: The Biopolitics of Pork in the United States, 1865–1930’, *American Quarterly*, 68.1 (2016), 49–73; Sandra Swart, ‘“It is as Bad to be a Black Man’s Animal as it is to be a Black Man” – The Politics of Species in Sol Plaatje’s *Native Life in South Africa*’, *Journal of Southern African Studies*, 40.4 (2014), 689–705.

8 Jonathan Saha, *Colonizing Animals* (Cambridge University Press, 2021), p. 25. Italics in original.

9 Saha broaches this very point in an earlier work while problematising the idea of breeds. See Jonathan Saha, ‘Milk to Mandalay: Dairy Consumption, Animal History and the Political Geography of Colonial Burma’, *Journal of Historical Geography*, 54 (2016), 1–12 (p. 9).

10 For a crop comparison, see Helen Anne Curry, ‘Taxonomy, Race Science, and Mexican Maize’, *Isis*, 12.1 (2021), 1–21.

11 For examples, see *Worlds of Natural History*, ed. by H. A. Curry, N. Jardine, J. A. Secord, and E. C. Spary (Cambridge University Press, 2018).

12 The Landwirtschaftliche Hochschule Berlin was soon thereafter incorporated into Humboldt University of Berlin. Hellmut Epstein, ’Fideikommiss-und Anerbenrecht in ihren Einflüssen auf Organisationsformen und Erträge der Landwirtschaf’ (unpublished doctoral thesis, Landwirtschaftliche Hochschule Berlin, 1928). Cited in *Bibliography on Land Utilization, 1918–36. USDA Miscellaneous Publication No. 284* (Washington: Government Printing Office, 1938), p. 947.

13 The fact that Raymond Dart made similar inferences from South Africa at the same time suggests a possibility that he and Epstein knew of each other through common acquaintances. Alexis Thomas was journal editor when Dart was asked to write about animal husbandry for the publication. Thomas was a professor of pathology in Onderstepoort where Epstein’s co-author Curson was also stationed.

14 Charles Darwin, *The Variation of Animals and Plants Under Domestication* (London: J Murray, 1868), p. 91.

15 Darwin, *The Variation*, p. 97.

16 See also, Francis Galton, ‘Gregariousness in Cattle and in Men’, *Macmillan’s Magazine*, 23 (1870), pp. 353–57.

17 Thanks to Gianamar Giovannetti-Singh for directing me towards this work. David Johnson, ‘Representing Cape “Hottentots,” From the French Enlightenment to Post-Apartheid South Africa’, *Eighteenth-Century Studies*, 40.4 (2007), 525–52 (p. 527); see also Lombard and Parsons, ‘Milk not Meat: The Role of Milk amongst the Khoe Peoples of Southern Africa’, *Journal of African Archaeology*, 13.2 (2015), 149–66; Elizabeth Sutton, ‘Mapping Meaning: Ethnography and Allegory in Netherlandish Cartography, 1570–1655’, *Itinerario*, 23.3 (2009), 12–42.

18 Clapperton Chakanetsa Mavhunga, ‘Vermin Beings: On Pestiferous Animals and Human Game’, *Social Text*, 29.1 (2011), 151–76. For a Black feminist critique that reclaims the humanism of animality while confronting its historical premise in the conception of humankind, see Zakiyyah Iman Jackson, *Becoming Human: Matter and Meaning in an Antiblack World* (NYU Press, 2020).

19 On the history of this classification, see Annemarie de Waal Malefijt, ‘Homo Monstrosus’, *Scientific American*, 219.4 (1968), 112–19.

20 Robert Gordon, ‘The Rise of the Bushman Penis: Germans, Genitalia and Genocide’, *African Studies*, 57.1 (1998), 27–54; Yvette Abrahams, ‘The Great Long National Insult: “Science”, Sexuality and the Khoisan in the 18th and early 19th century’, *Agenda*, 13.32 (1997), 34–48.

21 T. F. Gosset, *Race: The History of an Idea in America* (Oxford University Press, 1997), pp. 3–16; W. M. Evans, ‘From the Land of Canaan to the Land of Guinea: The Strange Odyssey of the “Sons of Ham”’, *The American Historical Review*, 85.1 (1980), 15–43; E. R. Sanders, ‘The Hamitic Hypothesis: Its Origin and Functions in Time Perspective’, *Journal of African History*, 10.4 (1969), 521–32.

22 Michael F. Robinson, *The Lost White Tribe: Explorers, Scientists, and the Theory that Changed a Continent* (Oxford University Press, 2016); Robin Derricourt, *Inventing Africa: History, Archaeology and Ideas* (Plutopress, 2011).

23 Jean Hiernaux, *The People of Africa* (London: Weidenfeld and Nicolson, 1974), p. 59.

24 Shomarka Keita, ‘Legacies in Science and Society of Charles Seligman’s Biocultural Theory of Africa (Hamitic hypothesis) and Ashley Montagu’s on Race’, *American Anthropologist*, 125.4 (2023), 797–808 (p. 799).

25 Solleveld, ‘Lepsius’, p. 202.

26 Drake, ‘The Responsibility’, pp. 228–43.

27 Guiseppe Sergi, *The Varieties of the Human Species: Principles and Method of Classification* (Smithsonian Institute, 1894); Guiseppe Sergi, *The Mediterranean Race: A Study of the Origin of European Peoples* (Walter Scott, 1901).

28 Peter Rohrbacher, ‘Hamitische Wanderungen’: Die Prähistorie Afrikas zwischen Fiktion und Realität’, in *Vom Wandern der Völker: Migrationserzählungen in den Altertumswissenschaften*, ed. by F. Wiedemann, K. P. Hofmann, and H.-J. Gehrek, Berlin Studies of the Ancient World, 41 (Humbodt-Universität zu Berlin, 2017), pp. 249–82.

29 This was a paraphrase of J. H. Greenberg in W. MacGaffey, ‘Concepts of Race in Historiography of Northeast Africa’, *The Journal of African History*, 7.1 (1966), 1–17 (p. 2).

30 This historical term, ‘Hamitic Longhorn’, is treated throughout the paper as a category to explore, not a fact to reify.

31 Epstein, *The Origin, Vol. I*, pp. 555–56. These Shorthorn cattle are not to be confused with the British breed by the same name.

32 J. H. R. Bisschop, ‘Parent Stock and Derived Types of African Cattle, with Particular Reference to the Importance of Conformational Characteristics in the Study of their Origin’, *South African Journal of Science*, 33 (1937), 852–870 (p. 864).

33 Georges Louis Leclerc de Buffon, *Buffon’s Natural History, Vol. 8, Containing a Theory of the Earth, A General History of Man, of the Brute Creation, and of Vegetables, Minerals, &c, &c*. (T. Gillet, 1807), p. 58.

34 Epstein, *The Origin, Vol. I*, pp. 506–07.

35 Despite the dated naming convention, *Bos taurus* and *Bos indicus*, the two breed to reproduce fertile offspring.

36 H. Epstein, ‘Descent and Origin of the Afrikander Cattle’, *Journal of Heredity*, 24.12 (1933), 448–62 (p. 462).

37 Epstein, *The Origin, Vol. I*, pp. 513, 519. See historical discussion in Marleen Felius, Marie-Louise Beerling, David S. Buchanan, Bert Theunissen, Peter A. Koolmees, and Johannes A. Lenstra, ‘On the History of Cattle Genetic Resources’, *Diversity*, 6 (2014), 705–50.

38 H. Epstein, ‘The Origin of the Africander Cattle, with Comments on the Classification and Evolution of Zebu Cattle in General’, *Zeitschrift für Tierzüchtung und Züchtungsbiologie*, 66.2 (1956), pp. 97–148 (p. 130).

39 Epstein, *The Origin, Vol. I*, p. 543.

40 George W. Stow, *The Native Races of South Africa: A History of the Intrusion of the Hottentots and Bantu into the Hunting Grounds of the Bushmen, the Aborigines of the Country* (Swan Sonnenschein & Co. Ltd, 1905), p. 2; A. E. Voss, ‘The Image of the Bushman in South African English Writing of the Nineteenth and Twentieth Centuries’, *English in Africa*, 14.1 (1987), 21–40 (p. 35).

41 Stow, *The Native Races*, p. 267.

42 For a review, see ‘The Cape Khoekhoe and Korana’ in Alan Barnard, *Hunters and Herders of Southern Africa: A Comparative Ethnography of the Khoisan Peoples* (Cambridge University Press, 1992), pp. 156–75.

43 Stow, *The Native Races*, pp. 267–69; I. Schapera, *The Early Cape Hottentots* (Van Riebeeck Society, 1933).

44 The term ‘Bushman’ was historically used in reference to hunter-gatherers in Southern Africa. Nowadays, it is more common to refer to these communities as San or the identity of their choosing.

45 Christos Lynteris, ‘Mahamari Plague: Rats, Colonial Medicine and Indigenous Knowledge in Kumaon and Garhwal, India’, *Medical Anthropology*, 41.4 (2022), 373–86; Clapperton Chakanetsa Mavhunga, *The Mobile Workshop: The Tsetse Fly and African Knowledge Production* (MIT Press, 2018); Helen Tilley, *Africa as a Living Laboratory: Empire, Development, and the Problem of Scientific Knowledge, 1870–1950* (University of Chicago Press, 2011).

46 John Lamphear, ‘The People of the Grey Bull: The Origin and Expansion of the Turkana’, *Journal of African History*, 29.1 (1988), pp. 27–39.

47 Lamphear, ‘The People’, p. 33.

48 He applied to same framework to explain Khoekhoe sheep, but not goats, which came later.

49 At first, Epstein traced Zebu origins in Central Asia to the progenitor species *Bos namadicus*. By 1971, he considered all cattle as a single species descending from geographical races of *Bos primigenius*.

50 Epstein, The Origin, *Vol. I*, pp. 482-488; Stow, *The Native Races*, p. 246.

51 Epstein, ‘Descent and Origin’, p. 462.

52 Epstein, ‘The Origin of the Africander’, p. 118.

53 Epstein, ‘The Origin of the Africander’, pp. 115–23; See comments by Sandra Swart in M. Derry, D. Haraway, D. Landry, H. Ritvo, S. Swart, K. Guest, and M. Mattfeld. ‘Humanimalia Roundtable on Breed’, *HUMaNIMALIA*, 10.1 (2018), pp. 5–26 (p. 16).

54 Pieter Labuschagne, ‘The Afrikaner cattle breed as a foundational symbol in Afrikaner culture: The legacy of Rooi Joseph Du Plessis’, South African Journal of Cultural History, 27.2 (2013), pp. 36–54. The symbolic power of livestock breeds abounds in nationalist narratives; for instance, Harriet Ritvo, ‘Race, Breed, and Myths of Origin: Chillingham Cattle as Ancient Britons’, *Representations*, 39 (1992), pp. 1–22; Catherine Nash, ‘Breed wealth: Origins, Encounter Value and the International Love of a Breed’, *Transactions of the Institute of British Geographers*, 4.5 (2020), 849–61.

55 Labuschagne, ‘The Afrikaner Cattle Breed’, pp. 37–38; T. G. W. Reinecke, ‘Africander Cattle’, *Journal of the Department of Agriculture*, 8.6 (1924), pp. 583–593 (p. 584).

56 A. C. MacDonald, ‘The Africander Breed of Cattle’, Transvaal Agricultural Journal, 3.9 (1904), pp. 1–4; Alex Holm, ‘Afrikander Cattle. Notes on their Origin, their Points, and their Qualities’, *South African Agricultural Journal* (1912) pp. 687–692.

57 H. Epstein, ‘Descent and Origin’, p. 461.

58 Charles S. Myers, ‘Charles Gabriel Seligman, 1873–1940’, *Obituary Notices of Fellows of the Royal Society, 1939–1941* <http://www.therai.org.uk/archives-and-manuscripts/obituaries/charles-gabriel-seligman> [accessed 21 September 2023]; Adam Kuper, *Anthropology and Anthropologists: The British School, 1922–1972* (Pica Press, 1973), pp. 1–24.

59 C. G. Seligman, *Races of Africa* (Thornton Butterworth Ltd, 1930).

60 C. G. Seligman, ‘Some Aspects of the Hamitic Problem in the Anglo-Egyptian Sudan’, *The Journal of the Royal Anthropological Institute of Great Britain and Ireland*, 43 (1913), 593–705 (pp. 654–58).

61 Voss, ‘The Image’, pp. 24–34.

62 Epstein, ‘Descent and Origin’, p. 462.

63 Dart, ‘Studies in Native Animal Husbandry’, p. 63.

64 H. H. Johnston, ‘A Survey of the Ethnography of Africa: And the Former Racial and Tribal Migrations in that Continent’, *The Journal of the Royal Anthropological Institute of Great Britain and Ireland*, 43 (1913), 375–421.

65 Epstein, *The Origin, Vol. I*, p. 495.

66 Ibid.

67 On this point, see David Arnold, ‘The Camera and the Cattle: Bovine Photography and Technologies of “Improvement” in Colonial South India’, *Technology and Culture*, 64.4 (2023), 1045–70.

68 Epstein, *The Origin, Vol. I*, p. 203.

69 Leopold Adametz, *Herkunft and Wanderungen der Hamiten, erschlossen aus ihren Haustierrassen* (Osten und Orient, 1920); A. Wiedemann, ‘Adametz Leop. Herkunft und Wanderungen der Hamiten, erschlossen aus ihren Haustierrassen (Osten und Orient,1920)’, *Indogermanische Forschungen*, 40.s1 (1922), 319–22 <http://dx.doi.org/10.1515/if-1922-0131>.

70 H. H. Curson and H. Epstein, ‘A Comparison of Hamitic Longhorn, West African Shorthorn and Afrikander Cattle, Particularly with Regard to the Skull’, Anatomical Studies No. 5, *Onderstepoort Journal of Veterinary Science and Animal Industry*, 3.2 (1934), 487–504 (p. 487).

71 Curson and Epstein, ‘A Comparison’, p. 487.

72 Epstein, ‘Descent and Origin’, pp. 457–58.

73 For an understanding of this doctrine in the history of science, see Nick Hopwood, *Haeckel’s Embryos: Images, Evolution, and Fraud* (University of Chicago Press, 2015).

74 Curson and Bisschop, ‘Some Comments’, p. 630.

75 H. H. Curson, ‘A Comparison of Two of the So-called Zebus’, Anatomical Studies, No. 61, *Onderstepoort Journal of Veterinary Science and Animal Industry*, 7.1 (1936), 371–91 (p. 372).

76 Neither vertebrae nor skull proved definitive for making the classificatory division within *Bos indicus*. Both groups displayed a convex skull and bifid spinous processes of the thoracic vertebrae.

77 H. H. Curson and R. W. Thornton, ‘A Contribution to the Study of African Native Cattle’, *Onderstepoort Journal of Veterinary Science and Animal Industry*, 7.2 (1936), 613–739 (p. 615). They dedicated the publication on African cattle to the memory of A. C. MacDonald, former Deputy-Director of Agriculture in Transvaal, ‘who first recognized the Afrikander as a native beast’. See A. C. MacDonald, ‘The Africander Breed of Cattle’, *Transvaal Agricultural Journal*, 3.9 (1904), pp. 1–4.

78 Curson and Thornton, ‘A Contribution’, p. 619.

79 Ibid., p. 620.

80 Ibid.

81 Epstein, ‘The Origin of the Africander’, p. 97.

82 Ibid., p. 102.

83 I. L. Mason, *The Classification of West African Livestock*, Technical Communication No. 7 of the Commonwealth Bureau of Animal Breeding and Genetics, Edinburgh. (Commonwealth Agricultural Bureaux, 1951), p. 7. Italics in original.

84 Mason argued that Sanga had implied an Afrikander influence and made no sense for Zebu crossed with West African Shorthorns. Mason, *The Classification*, p. 16; Epstein, *The Origin, Vol. I*, p. 339.

85 Bisschop, ‘Parent Stock’, p. 687.

86 Curson and Thornton, ‘A Contribution’, 636. Italics in original.

87 Curson and Epstein, ‘A Comparison’, p. 488.

88 Curson and Thornton, ‘A Contribution’, pp. 637–40.

89 W. J. A. Payne, ‘The Origin of Domestic Cattle in Africa’, *Empire Journal of Experimental Agriculture*, 32.126 (1964), pp. 97–113; on N’Dama, see Tad Brown, ‘Africa and the Cattle Without History’, *Global Environment*, 17.3 (2024), pp. 579–613.

90 Payne, ‘The Origin’, p. 99.

91 Curson and Epstein, ‘A Comparison’, p. 489.

92 Epstein, *The Origin, Vol. I*, pp. 203–04.

93 For scepticism about exaggeration in ancient Egyptian art, see Nicolle Leary, ‘Fit for the Job: Proportion and the Portrayal of Cattle in Egyptian Old and Middle Kingdom Elite Tomb Imagery’, *Arts*, 10.13 (2021), 13 <<http://dx.doi.org/10.3390/arts10010013>.

94 Epstein traces the social selection for large horns among Maasai cattle to Hamitic origins, too. H. Epstein, ‘Domestication Features in Animals as Functions of Human Society’, *Agricultural History*, 29.4 (1955), 137–46 (p. 139).

95 *Fulbe* was the traditional self-denomination (*Pulo* in singular). Fulani, a plural form of Fula coming through Arabic, became popular as the name used for them by neighbouring Hausa and, subsequently, European authors. Here, I use Fulani and Fula because of its common usage by those in West Africa today who identify themselves by the name and to maintain the benefit of orthographic consistency.

96 Seligman, *Races of Africa*, pp. 152–56.

97 A key source was G. P. Murdock, *Africa: Its Peoples and Their Culture History* (McGraw-Hill, 1959).

98 In Johnston’s opinion, ‘the general term “Fula” seems to me the best’ for the people and their language, as ‘*Pulo* is a Fula person, *Ful-be* the Fula race or people, and *Ful-ful-de* the language’. See H. H. Johnston, ‘The Fulas and Their Language’, *Journal of the Royal African Society*, 20.79 (1921), 212–16 (p. 213).

99 Captain T. G. de Guiraudon had intended to contribute a study but lost his copious fieldnotes, see T. G. de Guiraudon, ‘Notes and News’, *Journal of the Royal Asiatic Society*, 20.3 (1888), 459; see also, R. Basset. *Mission au Sénégal*, 3 vols (Paris, 1909–1910).

100 T. G. de Guiraudon, ‘Notes on African Philology’, *The Journal of the Royal Asiatic Society of Great Britain and Ireland*, 20.1 (1888), 145–46.

101 Obituary, ‘M.M. Delafosse’, *Nature*, 2988.119 (1927), 206; H. R. Palmer, ‘M. Delafosse’s Account of the Fulani’, *Journal of the Royal African Society*, 13.50 (1914), 195–203. This quote came from Delafosse’s three volume work, *Haut Senegal et Niger* (Paris, 1912).

102 Palmer, ‘M. Delafosse’s Account’, p. 195.

103 Ibid., p. 199.

104 G. H. Pirie, ‘Johnston, Henry Harry (1858–1927)’, in *Dictionary of Nineteenth-Century British Scientists*, vol 2, ed. by B. Lightman (Thoemmes, 2004), pp. 1088–93.

105 Johnston, ‘The Fulas’, pp. 215–16.

106 Ibid., p. 215.

107 H. R. Palmer, ‘The “Fulas” and their Language’, *Journal of the Royal African Society*, 22.86 (1923), pp. 121–30.

108 H. H. Johnston, ‘A Survey of the Ethnography of Africa: And the Former Racial and Tribal Migrations in that Continent’, *The Journal of the Royal Anthropological Institute of Great Britain and Ireland*, 43 (1913), 375–421 (p. 401).

109 J. H. Greenberg, ‘Studies in African Linguistic Classification: II. The Classification of Fulani’, *Southwestern Journal of Anthropology*, 5.3 (1949), 190–98 (p. 198); see also, G. P. Murdock, *Africa: Its Peoples and Their Culture History* (McGraw-Hill, 1959), p. 13.

110 Instead, Greenberg would classify Fula within the Sudanic family of Niger-Congo languages, consistent with a recent eastward migration from Senegambia. See Greenberg, ‘Studies’, p. 192. Genetic evidence continues to support the view that Fulani descended from the upper Nile, and not Eurasia. See Clyde Winters, ‘Fulani are not from the Middle East’, *PNAS*, 107.34 (2010), E132.

111 Sir H. H. Johnston, *The Opening Up of Africa* (Williams & Norgate Ltd, 1911).

112 Ibid., pp. 115–16.

113 Ibid., pp. 115–17.

114 Palmer, ‘M. Delafosse’s Account’, p. 202.

115 Epstein, *The Origin, Vol. I*, p. 498.

116 See R. A. Kennedy, ‘West Africa in Prehistory’, *History Today*, 8.9 (1958), 646.

117 Edmund D. Morel, *Affairs of West Africa* (William Heinemann, 1902), p. 151.

118 H. A. S. Johnston, *The Fulani Empire of Sokoto* (Oxford University Press, 1967).

119 C. Pierre, *L’Elevage dans l’Afrique Occidentale Francaise*, ed. Augustin Challemal (Librairie Maritime et Coloniale, 1906).

120 Morel, *Affairs*, p. 143; see also, Johnston, *The Opening*, p. 119, who claimed that the earliest Fula had possessed Zebu or Hamitic Longhorns.

121 Epstein, *The Origin, Vol. I*, pp. 552–554 (p. 554).

122 Ibid.

123 J. L. Stewart, ‘The Cattle of the Gold Coast’, *The Empire Journal of Experimental Agriculture*, 6.21 (1938), 85–94 (p. 92).

124 *Epstein, The Origin, Vol. I*, p. 549. Classification of Afrikander as Sanga appeared in I. L. Mason and J. P. Maule, *The Indigenous Livestock of Eastern and Southern Africa* (Commonwealth Agricultural Bureaux, 1960).

125 Epstein, *The Origin, Vol. I*, p. 556.

126 Frank A. Salamone, ‘Colonialism and the Emergence of Fulani Identity,’ *Journal of Asian and African Studies*, 20.3–4 (1985), 193–202 (p. 196).

127 A. L. Stoler, ‘Rethinking Colonial Categories: European Communities and the Boundaries of Rule’, *Comparative Studies in Society and History*, 31.1 (1989), 134–61; W. Beinhart and L. Hughes, *Environment and Empire* (Oxford University Press, 2007), pp. 1–21; Alan Lester, ‘Imperial Circuits and Networks: Geographies of the British Empire’, *History Compass*, 4.1 (2006), 121–41.

128 Karen Brown, ‘Tropical Medicine and Animal Diseases: Onderstepoort and the Development of Veterinary Science in South Africa 1908–1950’, *Journal of Southern African Studies*, 31.3 (2005), 513–29.

129 The rand donated from Hebrew University came at the initiative of a Justice of the Circuit Court in Jerusalem, Felix Landau, who was born in Transvaal.

130 Credit to Staffan Müller-Wille for making this point clear to me in conversation.

131 Birgitta Farelius, ‘“Where does the Hamite Belong?”‘, *Nomadic Peoples*, 32 (1993), 107–18.

132 On the need to confront the influence of the Hamitic hypothesis in various modes of thought, see Keita, ‘Tracing a Genealogy’, pp. 804–06; also, Saint-Clair Drake, ‘The Responsibility of Men of Culture for Destroying the “Hamitic Myth”’, *Présence Africaine*, 1 (1959), 228–43.

133 For recent analysis of the genetic signatures related to this pastoral history, see O. Hanotte, D. G. Bradley, J. W. Ochieng, Y. Verjee, E. W. Hill, and J. E. O. Rege, ‘African Pastoralism: Genetic Imprints of Origins and Migrations’, *Science*, 296 (2002), 336–39 <http://dx.doi.org/10.1126/science.1069878>; Felius et al., ’On the History’, 728; Marleen Felius, ‘On the Breeds of Cattle: Their History, Classification, and Conservation’, (unpublished doctoral thesis, Utrecht University, 2016), p. 414.

134 Excellent examples are found in works by Fiona Marshall, Elisabeth Hildebrand, and Anneke Janzen.

